# Association Between Serum G Protein–Coupled Estrogen Receptor and the Peripheral Blood Balance of T-Helper/New Effector T-Cells in Patients With Hashimoto's Thyroiditis

**DOI:** 10.1155/ije/1918396

**Published:** 2025-11-21

**Authors:** Xiu Zang, Wenruo Chen, Ran Liu, Yanhong Lin, Xuekui Liu, Houfa Geng, Jun Liang

**Affiliations:** ^1^Department of Endocrinology and Central Laboratory, Xuzhou Central Hospital, Xuzhou Institute of Medical Sciences, Xuzhou, Jiangsu, China; ^2^The Affiliated Xuzhou Clinical College of Xuzhou Medical University, Xuzhou, Jiangsu, China

**Keywords:** G protein–coupled estrogen receptor, Hashimoto's thyroiditis, Th17 cells, Treg cells, Treg/Th17 ratio

## Abstract

**Objective:**

To investigate the effects of the G protein–coupled estrogen receptor (GPER) on the balance of regulatory T-helper/new effector T-cells (Treg/Th17) in the peripheral blood of patients with Hashimoto's thyroiditis (HT) and healthy individuals.

**Methods:**

A total of 230 participants were enrolled in this study, including 206 patients with new-onset HT (HT group) and 24 healthy physical examinees (normal control [NC] group). Venous blood samples were obtained from the participants and tested for serum GPER levels using an enzyme-linked immunosorbent assay. The peripheral blood proportion of Treg and Th17 cells and the Treg/Th17 ratio were determined using flow cytometry. Thyroid function, antibody levels, and biochemical and anthropometric tests were performed. Data management and statistical analyses were performed using SPSS Version 25.0.

**Results:**

The serum GPER levels among the HT group participants were significantly higher than those among the NC group (*p* < 0.001). Among the HT group participants with increasing serum GPER levels, the peripheral blood proportion of Treg cells and the Treg/Th17 ratio increased significantly (*p* < 0.001), even after adjusting for relevant confounding factors. However, with increasing serum GPER levels, the peripheral blood proportion of Th17 cells decreased significantly (*p* < 0.001), even after adjusting for confounding factors.

**Conclusion:**

The results confirmed that the serum GPER expression level in the initial HT group was significantly higher than that in the NC group and was positively correlated with the Treg/Th17 ratio, peroxidase antibody, and thyroglobulin antibody. Our findings suggest that a compensatory increase in the proportion of Treg/Th17 cells may be related to increased serum GPER levels.

## 1. Introduction

Hashimoto's thyroiditis (HT) is an autoimmune thyroid disease in which immune tolerance breaks down due to the interplay of immunological, genetic, and environmental factors [1, 2].

In recent years, the incidence of HT has been increasing, with a noticeable trend toward younger populations, including children [3]. Approximately 50% of patients with HT, including children, pregnant women, and women of reproductive age, develop hypothyroidism [4], drawing significant clinical attention and necessitating further research into its pathogenesis.

Although HT is known to have a genetic predisposition, its exact etiology remains unclear [2]. Emerging evidence suggests that intestinal dysbiosis, bacterial overgrowth, and increased intestinal permeability contribute to the development of HT. Currently, the diet-microbiota–immune axis is considered an important factor in disease progression [5]. CD4+ T-cells play a central role in breaking immune tolerance. These cells differentiate into various subsets, including T-helper (Th) 1, Th2, Th17, and regulatory T-cells (Treg) [6–9].

Increasing evidence suggests that Th17 and Treg cells are involved in HT pathogenesis [10, 11], mainly through the Treg/Th17 imbalance theory [12]. Several studies have demonstrated that loss or functional defects in Treg are closely related to HT pathogenesis [13–16]. However, Bossowski et al. did not find a decrease in the proportion of Treg cells in patients with HT [17, 18]. Several studies have reported a decrease in the number or function of Treg cells along with an increase in Th17 cells in patients with HT, with Th17 levels correlating positively with disease severity [19–23]. These studies suggest that HT development may be associated with excessive Th17 cell activation [13]. However, Guo et al. found that the proportion of Th17 cells is reduced in patients with HT [18]. This indicates that immune dysregulation in HT is complex and has not yet been fully elucidated.

Recent studies have explained the possible causes of increased expression of peripheral blood Treg cells and/or decreased expression of Th17 cells as well as an imbalance in Th17/Treg cells in HT. However, the associated clinical and experimental phenomena remain unclear. Further research is required to explore the potential mechanisms underlying the immune pathogenesis of HT.

The G protein–coupled estrogen receptor (GPER) is a novel transmembrane nonclassical estrogen receptor identified in the late 1990s [24–26]. In 2005, Revankar et al. found that GPER expression was high in various immune cells, including B- and T-cells, monocytes/macrophages, and neutrophils [27, 28].

Recent studies have revealed that GPER plays an important role in regulating immune responses in autoimmune diseases, possibly by promoting the secretion of interleukin (IL)-10 and amplifying Treg cells, thereby inhibiting the Th17 cell–mediated immune response, which can be inhibited by activating the GPER-direct antagonist G15 and inhibiting the GPER agonist G1 receptors [29–32]. These studies have shown that GPER is closely associated with several autoimmune diseases. HT is a common organ-specific autoimmune disease, and GPER is expressed in the thyroid gland. However, whether GPER plays an important role in the immune pathogenesis of HT remains unclear. Currently, there are few reports on the correlation between the GPER and HT. Therefore, this study aimed to investigate the correlation between serum GPER levels and HT development and its potential mechanism of action.

## 2. Participants and Methods

### 2.1. Participants

A total of 230 participants aged 18–75 years were enrolled in the Xuzhou Clinical School of Xuzhou Medical University from July 2023 to February 2024. Of these, 206 patients with new-onset HT (HT group) and 24 healthy participants (normal control [NC] group) were included in the study. Informed consent was obtained from all patients. This study was reviewed and approved by the Ethics Committee of the Xuzhou Clinical School of Xuzhou Medical University (XZXY-LK-20230714-0116).

#### 2.1.1. HT Group Inclusion Criteria

The HT group inclusion criteria include the following:

1. A clinical diagnosis of HT based on increased serum levels of antibodies against thyroid peroxidase and/or thyroglobulin and ultrasound examination demonstrating a characteristic heterogeneous echotexture.
2. New-onset HT with normal or abnormal thyroid function.
3. Provision of signed informed consent.
4. Those with complete clinical information.

#### 2.1.2. NC Group Inclusion Criteria

The NC group inclusion criteria include the following:

1. Normal thyroid function and color ultrasonography.
2. Provision of signed informed consent.
3. Those with complete clinical information.

#### 2.1.3. HT and NC Group Exclusion Criteria

The HT and NC group exclusion criteria include the following:

1. Pregnant or postmenopausal women and women with irregular menstrual cycles.
2. Those receiving hormone replacement therapy, such as oral contraceptives.
3. Those with endocrine disorders, such as diabetes and polycystic ovary syndrome and those receiving drugs known to affect estrogen levels, such as calcium channel blockers and histamine-2 receptor antagonists.
4. Those with mental illnesses such as depression.
5. Those with other autoimmune diseases such as subacute thyroiditis, nodular goiter, or thyroid cancer.
6. Those with a history of thyroid cancer and/or surgical treatment.
7. Those with severe liver and kidney dysfunction, cardiac dysfunction, and other serious organ diseases.
8. Those with acute and chronic infections, immunodeficiency diseases, and tumors.
9. Those with an unclear history of the disease and incomplete questionnaire data.

### 2.2. Methods

#### 2.2.1. Data Collection

A professional staff member measured participants' height, weight, and blood pressure (BP). Height and weight were measured with patients wearing light clothing and without shoes. Body mass index (BMI) was calculated as weight (kg) divided by height squared (m). Trained doctors measured BP using a mercury sphygmomanometer on the dominant arm after a resting period of at least 5 min in the supine position. The patient's arm was placed at heart level, and BP values were taken as the mean of three measurements.

#### 2.2.2. Questionnaire Survey

Data on general condition, sex, age, history, family history, alcohol and tobacco habits, and medications used in the previous 6 months were obtained by qualified physicians and researchers and recorded on a unified designed form. All staff members were intensively trained in standard operational procedures and methods prior to the study.

### 2.3. Experimental Procedures

#### 2.3.1. Specimen Collection

A venous blood sample was drawn from all patients after an overnight fast (at least 10 h) and allowed to clot at room temperature for 1–3 h. Immediately after blood clotting, the serum was separated using centrifugation for 15 min at 3000 r/min (centrifugal radius, *r* = 11 cm). The supernatant was stored at −20°C or −80°C to prevent repeated freezing and thawing. The samples were sealed and stored.

#### 2.3.2. Measurement of General Biochemical Indicators

The levels of the following biomarkers were measured in all participants: white blood cells (WBC), red blood cells (RBC), platelets (PLT), fasting blood glucose (FBG), total cholesterol (TC), triglycerides (TG), low-density lipoprotein (LDL), high-density lipoprotein (HDL), serum creatinine, serum uric acid (SUA), aspartate aminotransferase (AST), alanine aminotransferase (ALT), and glutamyl transpeptidase (GGT). All biochemical assays were performed enzymatically using an autoanalyzer (Type 7600 Hitachi Ltd, Tokyo, Japan).

#### 2.3.3. Thyroid Function and Related Antibodies' Test

The levels of thyroid-stimulating hormone (TSH), free triiodothyroxine (FT3), free tetraiodothyroxine (FT4), peroxidase antibody (TPOAb), thyroglobulin antibody (TGAb), thyroglobulin (Tg), and TSH receptor antibody (TRAb) in the participants' serum were measured using an electrochemiluminescence immunoassay in the nuclear medicine radiology room of Xuzhou Central Hospital. The normal reference ranges are as follows: FT3, 2.63–5.7 pmol/L; FT4, 9.01–19.05 pmol/L; TSH, 0.35–4.94 uIU/mL; TGAb, 0–4.11 U/mL; TPOAb, 0–5.6 U/mL; TRAb, 0–1.75 U/L; and TG: 0–77 ng/mL.

#### 2.3.4. Serum GPER Level Measurement

The serum GPER level was measured using a commercial quantitative sandwich enzyme-linked immunosorbent assay (ELISA) kit (SEG045Hu; Cloud-Clone Corp., Houston, TX, USA) with a detection range of 0.312–20 ng/mL and a minimum detectable concentration of 0.113 ng/mL. There was no cross-reactivity with other related proteins, and the intra- and interassay variations were < 10% and < 12%, respectively.

The ELISA was performed as follows.

1. All standards, reagents, and samples were prepared before the experiment.
2. The standards or samples were added (100 μL) to each well and incubated at 37°C for 1 h.
3. Thereafter, 100 μL of Detection Reagent A working solution was added to each well, and the wells were covered with a plate sealer and incubated for 1 h at 37°C.
4. The plate was aspirated and washed thrice.
5. Thereafter, 100 μL of Detection Reagent B working solution was added to each well, covered with a plate sealer, and incubated for 30 min at 37°C.
6. The plate was aspirated and washed five times.
7. Thereafter, 90 μL of tetramethylbenzidine substrate solution was added to each well, covered with a new plate sealer, and incubated for 10–20 min at 37°C away from light.
8. Subsequently, 50 μL of Stop solution was added to each well. The liquid turned yellow after the addition of the Stop solution. Absorbance was measured at 450 nm using a microplate reader.
9. The sample concentrations were then determined.

The kit was stored at the recommended temperature until its expiration date. The same laboratory staff operated the kit according to the manufacturer's instructions under the same environmental conditions in the same laboratory.

#### 2.3.5. Measurement of the Proportion of Treg and Th17 Cells and the Peripheral Blood Mononuclear Cells (PBMCs) Using Flow Cytometry

##### 2.3.5.1. PBMC Separation

Peripheral venous blood (5 mL) was collected from the participants on empty stomach and coagulated with ethylenediaminetetraacetic acid. PBMCs were separated using Ficoll (*ρ* = 1.077) using density gradient centrifugation.

##### 2.3.5.2. Measurement of Th17 and Treg Cell Levels

From the participants, 100 μL of peripheral blood was collected in Tube A according to the experimental group, to which 100 μL of 1640 medium and 1 μL of the cell activation cocktail (with Brefeldin A) were added, placed in a 37°C incubator for 4 h, and gently shaken once every 30 min. After centrifuging Tube A, we discarded 100 μL of the supernatant, and the remaining 100 μL was resuspended. The experimental group collected another 100 μL of peripheral blood in Tube B. To Tubes A and B, 5 μL of CD3 Percp/Cy5.5 with CD4 FITC and 5 μL of CD4 FITC with CD25 APC were added, respectively. Tubes A and B were incubated in the dark for 10 min. Thereafter, 1 mL of RBC lysis buffer (1^∗^) was added to both tubes, which were kept in the dark for 15 min. After both tubes were centrifuged, the supernatants were discarded and the cells were resuspended in 1-mL PBS and recentrifuged. Subsequently, the supernatants were discarded, and 500 μL of fixation buffer was added for 15 min and thereafter recentrifuged. The supernatants were discarded, and the cells were washed with 1 mL of PBS. Next, 1 mL of intracellular staining perm wash buffer (1^∗^) was added to break the membrane by light treatment for 30 min, followed by centrifugation. Thereafter, the supernatants were discarded, and the cells were washed with 1 mL of PBS twice and resuspended in 100 μL of PBS. We then added 5 μL of IL-17 PE to Tube A and 5 μL of FOXP3 PE to Tube B for 15 min in the dark. The cells in Tubes A and B were washed twice with 1 mL of PBS by centrifugation, and the supernatant was discarded. Finally, 500-μL PBS was added to the cells for flow cytometry analysis.

The procedure was performed strictly according to the manufacturer's instructions. Each group of specimens was analyzed using a FACSCalibur flow cytometer. Data analysis was performed using Win MDI 2.9 software.

### 2.4. Statistical Analysis

Data management and statistical analyses were performed using SPSS Statistics for Windows, Version 25.0. All the hypotheses were tested using a two‐tailed tests. All data were normally distributed based on normality tests. Measured data are expressed as means ± standard deviation (*x* ± *s*). We used the propensity score method to match the data by matching the conditions of sex and age. In total, 21 cases were matched in each NC and HT groups. Independent sample *t*-tests were used to analyze differences in GPER levels and peripheral blood Treg/Th17 cell ratios after matching the two groups. In the HT group, we used the general linear equation test to analyze the correlation between GPER levels, thyroid autoantibody titers, and the Treg/Th17 ratio. Statistical significance was set at *p* < 0.05.

## 3. Results

### 3.1. Baseline Characteristics of Patients

A total of 230 participants were included in this study. Of these, 206 were clinically diagnosed with HT and had normal thyroid function. The participants were divided into HT and NC groups.  Table 1 presents the distribution of various indicators in the two groups.

### 3.2. Analysis of the Peripheral Blood Proportion of Treg and Th17 Cells, and the Ratio of Treg/Th17 in the HT and NC Groups

To determine the peripheral blood distribution of Treg/Th17 cells in the two groups, the data were matched using a biased scoring method. The final matching results included 21 age- and sex-matched individuals in the HT and NC groups as the observation groups. Among the participants in the matched-observation groups in the HT group, 11 were men and 13 were women, aged 25–50 years, with an average age of 31.57 ± 7.83 years, and in the NC group, 11 were men and 13 were women, aged 24–49 years, with an average age of 30.14 ± 7.84 years. No significant differences in sex composition or age were observed between the two groups (*p* > 0.05). Independent sample *t*-tests were used to analyze differences in GPER levels and peripheral blood Treg/Th17 cell ratios after matching the two groups. In the HT group, we used a general linear equation test to analyze the correlation between GPER levels and the Treg/Th17 ratio after matching the two groups.

Table 2 presents the differences between the peripheral blood proportions of Treg and Th17 cells and the Treg/Th17 ratio after matching. Peripheral blood Treg cell levels and Treg/Th17 ratio in patients with HT were significantly higher than those in healthy participants (*p* < 0.001), whereas the peripheral blood level of Th17 cells in patients with HT is significantly lower than that in healthy participants (*p* < 0.001).

### 3.3. Analysis of GPER Level, Thyroid Autoantibodies, and Thyroid Function in the HT and NC Groups

Table 3 presents the differences in GPER levels, thyroid antibodies, and functional indicators between the two groups. Serum GPER, TPOAb, and TGAb levels in patients with HT were significantly higher than those in healthy individuals (NC group) (*p* < 0.001, *p* < 0.001, and *p*=0.001, respectively).

### 3.4. Correlation Between GPER Level and Peripheral Blood Proportion of Treg and Th17 Cells and Treg/Th17 Cell Ratio

We further evaluated the correlation between GPER levels and peripheral blood proportions of Treg and Th17 cells, as well as Treg/Th17 cells. As presented in Table 4, in the absence of confounding factors, the proportion of Treg cells and Treg/Th17 ratio as dependent variables increased significantly with increasing GPER levels (*p* < 0.001), even after further adjustment for sex, age, FBG, systolic BP, diastolic BP, TGs, TC, HDL, LDL, and other interference factors (*p* < 0.001). However, as the dependent variable, the proportion of Th17 cells decreased significantly with increasing GPER levels (*p* < 0.001), regardless of whether the interference factors were adjusted.

### 3.5. Correlation Between GPER Level and Thyroid Autoantibodies

We further evaluated the correlation between serum GPER levels and thyroid autoantibodies including TGAb and TPOAb. As presented in Table 5, in the absence of confounding factors and with TGAb as the dependent variable, TGAb increased significantly with increasing GPER (*p*=0.012) even after adjusting for sex, age, FBG, systolic BP, diastolic BP, TGs, TC, HDL, LDL, and other interference factors (*p*=0.013). With TPOAb as the dependent variable, TPOAb changed significantly as GPER changed (*p* < 0.001), regardless of whether confounding factors were adjusted.

## 4. Discussion

The key mechanism underlying HT involves the breakdown of immune tolerance [33, 34]. Growing evidence indicates that Th17 and Treg cells play a role in HT pathogenesis, particularly through the Treg/Th17 imbalance theory [12]. Our study revealed that HT patients had a significantly higher proportion of Treg cells and a higher Treg/Th17 ratio in the peripheral blood than the NC group, while the Th17 cell proportion was significantly lower, consistent with previous reports by Brusko et al. [18, 35, 36]. Although some studies have suggested that HT progression may be linked to reduced Treg cell number or function and overactive Th17 cells [37–39], our findings showed the opposite trend in the peripheral blood. This discrepancy may be because Th17 cells migrate to the thyroid tissue, the main site of inflammation, thereby reducing their presence in the blood. Our findings are consistent with those of Guo et al., showing that during HT development, damaged Treg cells overcome or inhibit autoimmune responses through increased expression or functional activities. This negative feedback regulation causes a compensatory increase in Treg cells in the peripheral blood [36]. Some studies have suggested that this may represent a compensatory mechanism of the immune system for prolonging the immunopathological response [40]. However, our study did not explain the possible causes or mechanisms underlying the increased peripheral blood proportion of Treg cells in patients with HT.

Animal experiments and in vitro studies on GPER show that GPER is closely related to many autoimmune diseases. Few studies have examined the relationship between HT and GPER, as HT is an organ-specific autoimmune disease. In this study, we hypothesized that GPER inhibited Th17 cells by amplifying Tregs, causing a compensatory increase in the Treg/Th17 ratio. Therefore, they may play important roles in the pathogenesis of HT.

First, we analyzed the correlation between GPER and HT. Our results showed that serum GPER levels of patients in the HT group were higher than those in the NC group, and the difference was statistically significant. Recent studies have shown that GPER levels are elevated and the GPER agonist G-1 can reduce disease severity in animal models of experimental autoimmune encephalomyelitis and multiple sclerosis [29, 41]. These data suggest that G-1 is a novel therapeutic target for chronic autoimmune inflammatory diseases.

Moreover, some studies have shown that GPER is expressed in immune cells [41], and therefore its elevated serum levels in HT may be related to extensive lymphocyte infiltration in the thyroid. In contrast, many current studies have shown that G-1 can induce Foxp3 expression in cultured CD4+ T-cells, expand Treg cells, and promote IL-10 secretion, thereby inhibiting Th17 cell-mediated immune responses [29–32]. Moreover, the expression and/or function of Treg cells are reduced in patients with HT [42]. Therefore, we hypothesized that Treg cell damage may be regulated by negative feedback in HT, causing increased expression of GPER, thereby increasing the number of Treg cells, inhibiting Th17 cells, and causing a compensatory increase in the proportion of peripheral blood Treg cells to maintain the autoimmune balance, inhibit the inflammatory response, and reduce disease severity. However, their compensatory ability is limited. This imbalanced compensation results in disease progression. Therefore, we believe that this compensatory mechanism may be self-protective in the autoimmune system.

To explore our hypothesis, we determined the correlation between GPER levels and the Treg/Th17 ratio. We observed a dynamic correlation between GPER levels and the Treg/Th17 ratio. Similar to other studies [28, 30–32, 43], our results suggest that GPER promotes Treg expansion and suppresses Th17 cells, leading to an increased Treg/Th17 ratio in HT patients relative to healthy controls.

We also analyzed the relationship between GPER levels and thyroid autoantibodies. As expected, TPOAb and TGAb levels were significantly higher in the HT group. Moreover, serum GPER levels positively correlated with TPOAb and TGAb levels. Based on these findings, we propose that GPER may help regulate the Treg/Th17 balance, thereby modulating abnormal T-cell immune responses and reducing autoimmune damage to the thyroid tissue.

This study has some limitations. First, the study had a limited number of sources. Second, the sample size was small; therefore, further multicenter studies with larger samples are needed. Third, we did not analyze the relationship between GPER levels and HT duration. Finally, this study failed to investigate the relevant mechanisms underlying the cytological and molecular genetics of thyroid tissue specimens. As this was a cross-sectional study, animal models and prospective experiments are required for further investigation. These limitations may have affected our results.

## 5. Conclusion

We found an imbalance in the peripheral blood proportion of Treg and Th17 cells in patients with HT and a compensatory increase in the Treg/Th17 ratio in the early stages of HT. Furthermore, serum GPER levels were high in patients with HT. During HT pathogenesis, GPER levels dynamically change, correlating positively with the thyroid autoantibody titer and negatively with the peripheral blood Treg/Th17 ratio. This compensatory increase in the Treg/Th17 ratio may be related to the elevated expression of GPER. Therefore, GPER may inhibit the immune response by regulating the immune function imbalance of Treg/Th17 cell subsets during HT pathogenesis and participating in HT development. This study provides sample-based medical evidence for the prevention of hypothyroidism and treatment of HT and insights into drug development and targeted drug use.

## Figures and Tables

**Table 1:** Baseline characteristics of participants.

Variable	HT group	NC group
*N*	206	24
Male	40	11
Age (years)	33.5 ± 9.61	30.13 ± 7.47
GPER (ng/mL)	3.54 ± 2.86	0.64 ± 0.49
FT3 (pmol/L)	6.48 ± 6.69	5.36 ± 0.77
FT4 (pmol/L)	16.01 ± 8.51	17.55 ± 2.09
TSH (mIU/L)	6.15 ± 15.62	2.64 ± 2.17
TGAB (IU/ML)	299.09 ± 349.40	12.44 ± 6.89
TPOAB (IU/ML)	562.70 ± 375.18	12.54 ± 6.89
Tg (ng/mL)	25.07 ± 37.21	11.24 ± 5.85
TRAB (IU/L)	7.59 ± 11.23	0.43 ± 0.19
DBP (mmHg)	117.08 ± 11.39	118.55 ± 10.92
SBP (mmHg)	72.81 ± 7.01	76.0 ± 6.70
FBG (mmol/L)	5.23 ± 0.69	4.93 ± 0.57
TC (mmol/L)	3.96 ± 1.33	4.37 ± 0.42
TG (mmol/L)	1.49 ± 1.45	1.22 ± 0.86
HDL (mmol/L)	1.15 ± 0.32	1.32 ± 0.32
LDL (mmol/L)	2.39 ± 1.03	2.43 ± 0.55
SUA (umol/L)	313.21 ± 79.27	345.78 ± 114.54
Scr (umol/L)	40.69 ± 14.84	71.67 ± 11.64
AST (U/L)	20.86 ± 9.68	19.89 ± 5.53
ALT (U/L)	22.33 ± 16.42	21.33 ± 10.72
GGT (U/L)	22.05 ± 10.20	26.44 ± 37.49
WBC (×10^9^/L)	6.26 ± 1.93	5.72 ± 1.80
RBC (×10^12^/L)	4.59 ± 0.49	4.91 ± 0.56
PLT (×10^9^/L)	249.53 ± 65.46	227.0 ± 65.06

*Note:* ALT: glutamic pyruvate; AST: glutamic transaminase; FT3: free triiodothyroxine; FT4: free tetraiodothyroxine; GGT: glutamyl transpeptidase; GPER: G protein–coupled estrogen receptor; PLT: platelet; SBP: systolic pressure; Scr: serum creatinine; TG: triglycerides; Tg: thyroglobulin; TGAb: thyroglobulin antibody; TPOAb: peroxidase antibody; TRAb: thyroid-stimulating hormone receptor antibody.

Abbreviations: BMI = body mass index, DBP = diastolic blood pressure, FBG = fasting blood glucose, HDL = high‐density lipoprotein, LDL = low‐density lipoprotein, RBC = red blood cell, SUA = serum uric acid, TC = total cholesterol, TSH = thyroid-stimulating hormone, and WBC = white blood cell.

**Table 2:** Analysis of the peripheral blood Treg and Th17 cell levels and Treg/Th17 ratio in the HT and NC groups.

	NC group	HT group	*t* value	*p* value
*N*	21	21		
Age (years)	30.14 ± 7.84	31.57 ± 7.83	−0.591	0.558
Treg (%)	3.27 ± 0.45	3.87 ± 0.69	−3.615	< 0.001
Th17 (%)	9.22 ± 0.17	7.54 ± 1.50	5.41	< 0.001
Treg/Th17	0.36 ± 0.06	0.55 ± 0.21	−4.358	< 0.001

*Note:* Treg%, proportion of regulatory T-helper cells; Th17%, proportion of new effector T-cells. Treg/Th17, the ratio of regulatory T-helper cells to new effector T-cells.

**Table 3:** Analysis of GPER level and thyroid function in the HT and NC groups.

	NC group	HT group	*t* value	*p* value
*N*	21	21		
GPER (ng/mL)	0.65 ± 0.52	3.55 ± 2.75	−4.923	< 0.001
Age (years)	30.14 ± 7.84	31.57 ± 7.83	−0.591	0.558
FT3 (pmol/L)	5.33 ± 0.81	4.99 ± 1.74	0.824	0.415
FT4 (pmol/L)	17.45 ± 2.17	14.37 ± 5.09	2.559	0.014
TSH (mIU/L)	2.56 ± 2.26	5.33 ± 11.67	−1.069	0.292
TGAb (IU/ML)	12.67 ± 7.19	373.56 ± 400.87	−4.125	0.001
TPOAb (IU/ML)	12.65 ± 7.19	505.52 ± 361.52	−6.246	< 0.001

*Note:* FT3: free triiodothyroxine; FT4: free tetraiodothyroxine; TGAb: thyroglobulin antibody; TPOAb: peroxidase antibody.

Abbreviation: TSH = thyroid-stimulating hormone.

**Table 4:** Correlation between GPER and the proportion of Treg, Th17, and Treg/Th17 cells.

	*β*	*β* 95% CI	*t* value	*p* value
Treg (%)				
Model 1	0.519	0.353–0.684	6.317	< 0.001
Model 2	0.521	0.49–0.593	21.23	< 0.001
Th17 (%)				
Model 1	−1.148	−(1.469–0.826)	−7.175	< 0.001
Model 2	−1.213	−(1.328–1.098)	−21.24	< 0.001
Treg/Th17				
Model 1	0.156	0.113–0.199	7.271	< 0.001
Model 2	0.148	0.134–0.162	−20.04	< 0.001

*Note:* Model 1: unadjusted. Model 2: adjusted for sex, age, fasting blood glucose (FBG), systolic pressure (SBP), diastolic blood pressure (DBP), triglyceride (TG), total cholesterol (TC), high‐density lipoprotein (HDL), and low-density lipoprotein (LDL). GPER, G protein–coupled estrogen receptor; Treg (%), the proportion of regulatory T-helper cells; Th17 (%), the proportion of new effector T-cells; Treg/Th17, regulatory T-helper cells/new effector T-cells.

**Table 5:** Correlation between GPER, TGAb, and TPOAb.

	*β*	*β* 95% CI	*t* value	*p* value
TGAb				
Model 1	52.41	12.064–92.75	2.625	0.012
Model 2	53.66	11.98–95.33	2.607	0.013
TPOAb				
Model 1	79.11	40.23–117.99	4.112	< 0.001
Model 2	81.06	41.88–120.23	4.189	< 0.001

*Note:* TGAb: thyroglobulin antibody; TPOAb: peroxidase antibody; GPER: G protein–coupled estrogen receptor; Model l: unadjusted; Model 2: adjusted for sex, age, fasting blood glucose (FBG), systolic pressure (SBP), diastolic blood pressure (DBP), triglyceride (TG), total cholesterol (TC), high‐density lipoprotein (HDL), and low-density lipoprotein (LDL).

## Data Availability

The data that support the findings of this study are available on request from the corresponding author. The data are not publicly available due to privacy or ethical restrictions.
